# Developing a Functional Geopolymer Foam Composite from Natural Fibers and Kaolinitic Soil from Al-Kharj and Exploring Its Application in Water Treatment

**DOI:** 10.3390/ma19204356

**Published:** 2026-10-16

**Authors:** Mazen Alshaaer, Bader Alharbi, Obaid Alqahtani, Mohammed S. Alotaibi, Abdullah Fahad Alzayed

**Affiliations:** Department of Physics, College of Science and Humanities in Al-Kharj, Prince Sattam Bin Abdulaziz University, Al-Kharj 11942, Saudi Arabia; ba.alharbi@psau.edu.sa (B.A.); om.alqahtani@psau.edu.sa (O.A.); m.alotiaby@psau.edu.sa (M.S.A.); a.alzayed@psau.edu.sa (A.F.A.)

**Keywords:** wollastonite, geopolymers, metakaolin, foam composite, Al-Kharj

## Abstract

In recent decades, researchers have developed innovative compositions and methods to fabricate geopolymers and enhance their properties for diverse multifunctional applications. Geopolymer foams are regarded as promising functional materials across numerous disciplines; however, they face challenges such as brittleness and limited mechanical strength. This investigation aims to produce a geopolymer foam composite by incorporating three-dimensional (3D) Luffa Cylindrical Fibers (LCF) into a metakaolin-wollastonite geopolymer matrix. The composites were synthesized using an alkaline activator, mineral powders (wollastonite and metakaolin), a foaming agent (aluminum powder), and a foam stabilizer (olive oil). Their mechanical characteristics and capacity for Cu and As ion adsorption were examined, with structural analyses supplemented by scanning electron microscopy (SEM) and optical microscopy. Reinforcing with LCF markedly improved mechanical performance: flexural strength increased from 0.27 MPa to 0.60 MPa, while compressive strength rose from 0.04 MPa to 0.34 MPa. In addition to the amorphous structure, two new crystalline phases were detected: analcime and anorthite in the GLF matrix. The geopolymer foam composite (GLF) exhibited adsorption capacities of 4.6 mg/g for Cu, 5.18 mg/g for As, and 5.09 mg/g for methylene blue (MB) dye. The equilibrium adsorption capacity (qe) for Cu ions varies with the GLF mass, with smaller GLF masses yielding higher qe. Specifically, qe increases from 2.1 mg/g to 12 mg/g as the GLF mass decreases from 2 g to 0.25 g. Visual assessments reveal enhanced dye adsorption following LCF incorporation. Its high efficacy in removing Cu ions and metal-uptake capacity underscores its potential for practical water-treatment applications, offering sustainable solutions for environmental management.

## 1. Introduction

Geopolymers are valued for their ability to harden at low temperatures, their versatility across applications, and their strong mechanical properties. They are used in various applications, including construction and waste recycling [1], water treatment [2], hazardous waste stabilization [3], and passive cooling systems [4]. However, using too much alkaline solution in the field can lead to technical problems, such as the need for large quantities of aggressive alkali solutions. Additionally, their higher cost compared to traditional cement makes widespread use challenging. Therefore, researchers are actively seeking ways to develop functional geopolymers as sustainable, eco-friendly materials for engineering and environmental uses, aiming to overcome these obstacles [5,6].

The combined characteristics of geopolymers and porous structures have prompted extensive research into their properties [7]. Factors such as porosity, pore type (closed or open), connectivity, and size distribution significantly affect their performance [8]. Typically, higher porosity reduces compressive strength, thermal conductivity, and bulk density [9]. The impact of pore type varies; fine, uniform closed pores often enhance mechanical strength and insulation more effectively than open pores. Elevated open porosity increases pore connectivity, producing larger, interconnected pores. These networks increase surface area and volume capacity, thereby enhancing water and sound absorption, though they may compromise structural strength. Pore size distribution is also significant; larger pores may weaken the material but can improve thermal insulation at higher porosity levels [10]. Generally, foams with lower pore connectivity exhibit higher strength and lower thermal conductivity, making them suitable for construction [11], thermal barriers [12], and sound insulation [13]. Greater pore connectivity increases porosity and surface area, broadening applications in wastewater treatment, including heavy metal adsorption, organic pollutant removal, and functional composite development. In conclusion, controlling and optimizing the pore structure in geopolymer foams remains central to ongoing research.

Interest in geopolymer foams for water treatment has surged recently [14,15,16]. Pore-forming agents can increase pore volume and improve pore structure [14]. Utilizing waste-derived geopolymers as affordable, eco-friendly adsorbents presents a promising strategy for industrial wastewater treatment and sustainable production [17]. Geopolymers used for cation exchange are a feasible alternative to traditional activated carbons [18]. Because research on geopolymer foam’s ability to adsorb micropollutants is limited, this review focuses on geopolymer materials, whether as solid blocks or powders, for this purpose. Recent reviews [15,19] often overlook the differences between bulk and powdered types. Lead adsorption has been observed at 81 mg/g with coal fly ash powders, while Cheng et al. achieved nearly 100 mg/g using metakaolin powders [20]. Liu et al. synthesized a floatable, permeable geopolymer block for dye extraction, which required grinding and sieving before use [21]. Powdered geopolymers can rapidly remove methylene blue (about 51 mg/g) but are difficult to recover or directly deploy in packed beds. Recently, monolithic forms such as membranes have gained interest as safer, more manageable alternatives that enable direct use in packed beds without additional support. For example, Ge et al. used a metakaolin-based cylindrical geopolymer membrane to extract Ni^2+^ from synthetic wastewater [22], while Novais et al. employed cylindrical discs to remove lead [23]. Although monoliths generally have lower adsorption capacities (0.95 to 6.3 mg/g) than powders [23], these studies are preliminary and focus primarily on bulk adsorbents [24]. Another bulk geopolymer achieved a maximum capacity of 37. 9 mg/g [25]. A cylindrical, mesoporous adsorbent with a surface area of 56.6 m^2^/g and 20 nm pores has been used for dye removal, achieving capacities of 46.6 mg/g for basic violet and 46.4 mg/g for malachite green oxalate. Foamed geopolymer monoliths were utilized to remove copper and ammonium, achieving removal efficiencies of 87% and 95%, with uptakes of 0.54 and 0.57 mg/g, respectively [15]. Tang et al. [16] described metakaolin geopolyloid spheres with a surface area of 54 m^2^/g and 60% porosity, which showed affinity for Cu^2+^, Pb^2+^, and Ca^2+^, with maximum uptakes of 35, 46, and 24 mg/g.

Geopolymer foaming involves generating and stabilizing gas bubbles in a reactive aluminosilicate slurry before hardening, primarily through chemical or physical foaming [26]. Chemical foaming produces gas in situ via reactions, such as hydrogen peroxide decomposing to oxygen and water, or metallic powders like aluminum reacting with alkaline solutions to release hydrogen [27]. Balancing gas release and geopolymerization prevents bubble collapse or coalescence. Physical foaming uses mechanical agitation and surfactants to entrain air, forming a foam that stabilizes as the gel forms. Final pore structure depends on gas kinetics, geopolymerization rate, slurry viscosity, activator, Si/Al ratio, water, curing temperature, and mixing [28]. Studies show that foaming performance varies with the route and stabilization; water evaporation yields dense materials, while metallic powders like zinc produce low-density, uniform pores. Different foaming strategies influence pore morphology and strength. Stabilizers like calcium stearate improve bubble stability, resulting in homogeneous pores and better thermal and mechanical properties [29]. Combining H_2_O_2_ with stabilizers such as silica fume or natural zeolite enhances thermal insulation [30]. In summary, geopolymer foam performance depends on the interaction between gas release, bubble stabilization, and gel setting. Achieving effective pore design requires careful coordination of bubble formation, expansion, and matrix hardening. For adsorption-focused systems, the primary goal is to enhance accessible surface area and pore connectivity while maintaining structural strength, ensuring that the foamed materials function effectively in flowing water and continuous capture applications. Geopolymers are attractive for adsorption due to their chemical stability, customizable porosity, strength, and shape flexibility. Their negatively charged aluminosilicate structure attracts cations, and they can be made into monoliths, foams, spheres, and other practical forms beyond powders for water treatment. These qualities make geopolymers promising for functional adsorbents, though their initial effectiveness is limited by low surface area and fewer active sites [31].

As with aerated concrete, additives can improve geopolymer foam concrete properties. Fibers, particularly syntactic ones such as polypropylene, polyvinyl alcohol, and basalt, enhance mechanical performance [32,33,34]. However, studies on natural fibers such as abaca or hemp remain limited [35,36]. Some researchers use natural fibers like hemp or abaca to reinforce geopolymer foam concrete. Researchers studied lightweight geopolymer concrete reinforced with hemp fibers for insulation. They made the hemp-based geopolymer by adding a hemp fiber grid to a slurry of metakaolin and sodium silicate. Silicon metal powder and vegetable surfactants produced the foam [35]. SEM images show the fracture surface, where high alkalinity chemically treats the surface and ensures strong adhesion between the matrix and fibers. Higher magnifications reveal excellent bonding due to fiber surface etching. Ngo et al. used hydrogen peroxide as a foaming agent and abaca fibers for reinforcement, with compressive strengths of 19.56 to 36.85 MPa and flexural strengths of 2.145 to 6.254 MPa [37]. Triwulan et al. studied fly ash-based geopolymer with abaca fibers at 0.4, 0.6, and 0.8 wt. %, finding that increasing fibers up to 0.8 wt. %, raises compressive strength to 3.05 MPa and reduces porosity from 61% to 57.5% [35]. Fiber-reinforced geopolymer foam concrete could replace traditional thermal insulators but faces challenges, especially with natural fibers. Variability in fiber quality, composition, and treatment complicates comparisons across studies. Enhancing interfacial bonding prevents fiber pullout, but increasing foam stability raises surface energy and risks destabilization.

A novel functional geopolymer foam has been introduced that can achieve high micropollutant adsorption [38,39]. However, these foams showed limited mechanical stability, with a compressive strength of 0.067 MPa, rendering them unsuitable for long-term immersion and turbulent water flow [38]. This study focuses on developing a reinforced geopolymer foam composite incorporating vascular LCFs and a novel metakaolin-wollastonite geopolymer matrix [39]. To the best of the authors’ knowledge, this is the first investigation to use continuous vascular LCFs as reinforcement in geopolymer foam. LCFs have a distinctive cellular structure that enables foaming and vascular characteristics, which are important for applications such as water treatment and passive cooling systems. LCF is derived from mature, dried sponge gourd fruit. With its unique three-dimensional porous network, it serves as a sustainable and environmentally friendly alternative to synthetic plastics, foams, and glass fibers across various green industries. The proposed foam composite combines desirable mechanical strength, low saturated bulk density, and efficient micropollutant removal. A noteworthy feature of these foam composites is their ability to remain afloat while maintaining substantial mechanical integrity, with a saturated bulk density below 1 g/cm^3^. This attribute makes them suitable for transport by water currents in ponds, rivers, lakes, seas, and reservoirs, facilitating large-scale water treatment.

## 2. Materials and Methods

### 2.1. Materials and Chemicals

**Figure 1:**
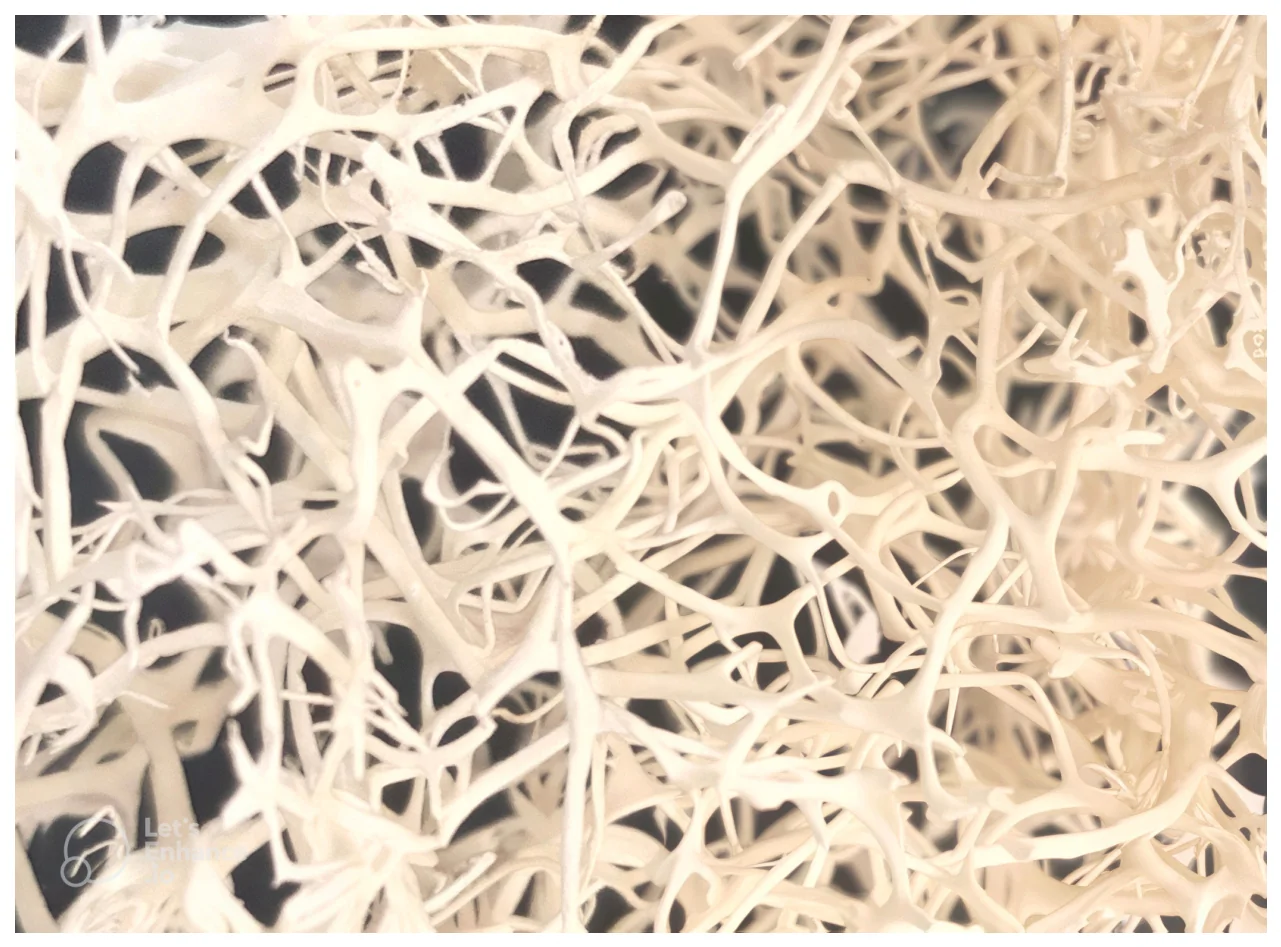
Elongated 3D LCFs.

The components used to synthesize the geopolymer foam composite included kaolinitic clay, wollastonite, a Na_2_SiO_3_ solution, and NaOH. A soil sample rich in kaolinite was collected from a deposit in Al-Kharj, Saudi Arabia, and laboratory analysis estimated its kaolinite content at approximately 85% based on loss-on-ignition measurements. According to ASTMD422 [40], the grain-size distribution indicates that the kaolinitic soil sample is predominantly fine-grained, with 87.75% consisting of fine sand and clay. The remaining 12.25% comprises coarse-grained soil, including various sand sizes. The soil was heated at 750 °C for 4 h in a Nabertherm furnace (Lilienthal, Germany) to produce amorphous kaolinite (metakaolin). Wollastonite (CaSiO_3_) sourced from NYCO (Paris, France) served as a secondary precursor. The median particle size of wollastonite ranged from 9 to 14 µm. An alkaline activator was prepared by mixing Na_2_SiO_3_ (27 wt.% SiO_2_ and 8 wt.% Na_2_O), NaOH, and distilled water. Deionized water and pure NaOH pellets (Merck, Germany) were used to prepare the NaOH solution. Fine aluminum powder (99.5% purity, ASSEEL Trading Corporation, Riyadh, Saudi Arabia) served as a foaming agent, while organic olive oil (Aljouf, Saudi Arabia) stabilized the foam. The luffa sponge was crafted from dried Luffa Cylindrical Fiber (LCF) fruit, using only the outer core as a natural fiber mat. This homogeneous outer core was cut into 3D fiber blocks as shown in Figure 1. The fiber mats were washed with distilled water and dried at 80 °C for 24 h. Each mat features two sides: one with unidirectional fibers and the other with randomly arranged fibers [5].

### 2.2. Synthesis of the Geopolymer Foam Composite

Solutions containing sodium silicate, sodium hydroxide, and metakaolin/wollastonite powder were prepared with molar ratios of Si/Al/Na set at 2:1:1. The resulting alkaline solution had an H_2_O/Na_2_O molar ratio of 6:1. To prepare this solution, NaOH, H_2_O, and Na_2_SiO_3_ were stirred continuously for 6 h. Then, metakaolin/wollastonite powder [40] was added to the alkaline solution, and the mixture was blended thoroughly with a mechanical mixer. After five minutes of mixing, olive oil (a stabilizer) and aluminum powder were added, followed by an additional thirty seconds of stirring. The 3D LCF (Figure 1) was elongated in three dimensions, then trimmed to fit the mold. The geopolymer mixture was poured into silicone molds, filling about one-third of each mold.

The molds were cylindrical—25 mm diameter and 50 mm height—for compressive strength tests, and rectangular—20 mm × 20 mm × 120 mm—for flexural tests. After 60 min at room temperature, the molds were sealed and cured in an oven (Raypa, Terrassa, Spain) at 40 °C for 24 h. Once demolded, the geopolymer foam composites were washed with distilled water for 6 h to remove residual salts and geopolymer foam (GLF). The samples were then characterized using various techniques. Control samples of geopolymer foam (GF) were prepared similarly but without including the 3D LCF.

Table 1 shows the compositions of the GLF and fiber-free GF. SiO_2_ sources include the alkaline solution and metakaolin. Sodium oxide (Na_2_O) came from the alkali activator solutions, and aluminum oxide (Al_2_O_3_) came from metakaolin. The proposed precursor in this study, wollastonite, has the potential to release both CaO and SiO_2_ during the geopolymerization process [41].

### 2.3. Characterization Techniques

The specimens’ morphology and microstructure were examined using gold-coated Scanning Electron Microscope (SEM) imaging (Quanta Inspect F50, FEI Company, Eindhoven, The Netherlands). Energy-dispersive X-ray spectroscopy (EDS) was coupled with the SEM. The samples underwent phase analysis using a Rigaku Ultima IV XRD diffractometer-6000 (Rigaku Corporation, Tokyo, Japan) with a 2-theta scanning range of 5–80° at a scan rate of 2°/min. MATCH! Software (Version 4, Crystal Impact, Bonn, Germany) was used to perform Rietveld refinement of the produced materials.

The compressive and flexural strengths of the samples were measured in accordance with ASTM C39 [42] and ASTM C78/C78M [43], respectively. The tests were conducted at room temperature on three specimens from each series using a universal testing machine (HD-B615-S, China). Three samples from each series were analyzed to establish their flexural and compressive strengths.

The compressive strength was calculated using the following equation:

$$\sigma_{c}=\frac{F}{A}$$

where σ_c_ is the compressive stress, F is the loading force, and A is the cross-sectional area. The samples are cylindrical, with a diameter of 25 mm and a height of 50 mm.

The flexural strength was calculated using the following equation for a three-point bending test:

$$\sigma_{f\;}=\;\frac{3FL}{2bd^{2}}$$

where σ_f_ is the flexural stress, F is the axial force at the fracture point, b is the sample width (20 mm), and d is the specimen thickness (20 mm). L (80 mm) is the distance between the supports, and the sample length is approximately 120 mm.

Bulk density is typically measured in accordance with ASTM C642 [44], which defines it as the specimen mass divided by volume. The open porosity was measured in accordance with ASTM C20 [45]. To evaluate the adsorption capacity of the geopolymer foams, we immersed cylindrical samples (~0.8 g) from each formulation in standard solutions (100 ppm/L) of Cu and As at pH 5, adjusted with NaOH/HClO_4_. After 12 h of shaking at 200 rpm, 10 mL samples were filtered through 0.45 μm nylon filters, centrifuged, and analyzed for ion concentrations using Inductively Coupled Plasma Optical Emission Spectroscopy (ICP-OES) (Thermo Fisher Scientific, Waltham, MA, USA, iCAP 7000 series).

A 4 × 10^−5^ M methylene blue solution was prepared in a 1000 mL volumetric flask to monitor dye adsorption visually. The sealed flask was agitated at 25 °C and 200 revolutions per minute for 2 h. Subsequently, 200 mL aliquots of this MB solution were distributed into three separate flasks, each containing an 8 g sample of geopolymer foam (GF or GLF) and a control. The color intensity of each solution was monitored at 2 h intervals until the GLF solution became clear (pure water).

The removal percentage (R%) and adsorption capacity (qe) of Cu ions were calculated utilizing Equations (1) and (2), respectively [46].

(1) $$R\%=\frac{C_{0\;}-C_{e}}{C_{0}}$$

(2) $$q_{e}=\frac{V}{m}\;(C_{0}-C_{e})$$

where C_0_ and C_e_ represent the initial and final solution concentrations (mg/L), respectively; v is the solution volume (L); and m is the mass of the adsorbent (g). q_e_ (mg/g) indicates the amount of adsorbate per unit mass of adsorbent at equilibrium.

## 3. Results and Discussion

### 3.1. The Effect of Natural Fibers on the Microscopic Features of Geopolymer Foam

**Figure 2:**
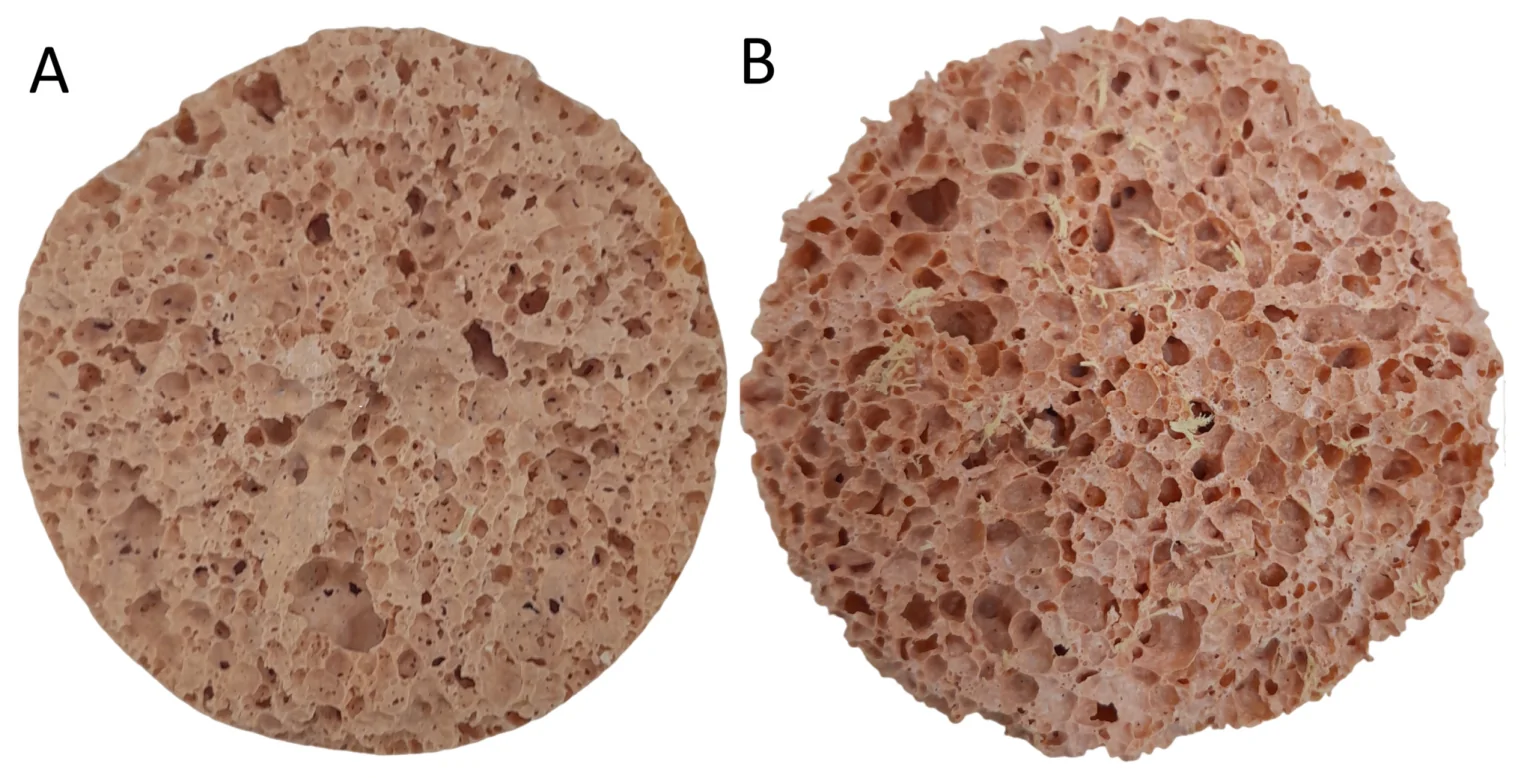
Geopolymer foam (**A**) and geopolymer foam composite reinforced with LCF (**B**).

GF were fabricated using a metakaolin-wollastonite matrix, as shown in Figure 2A. Adding the LCF network as reinforcement produced GLF, as shown in Figure 2B. Both foam types display a clear pore network spread throughout the matrix. Unlike traditional continuous fibers, the 3D LCF mat allows the geopolymer paste to expand uniformly during setting. By extending the LCF in three dimensions and maintaining appropriate fiber spacing (see Figure 1), the geopolymer foam develops a cohesive, well-organized network.

**Figure 3:**
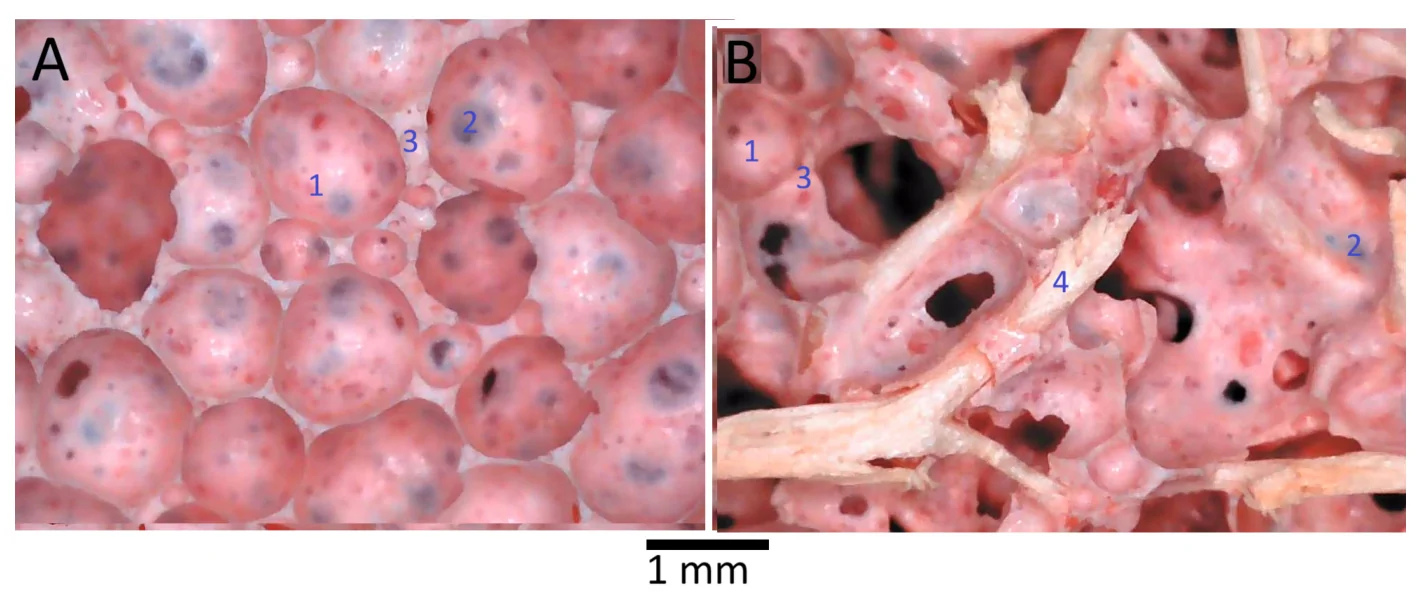
Images of geopolymer foam taken with an optical microscope: (**A**) GF and (**B**) GLF; (1) open pores, (2) cell wall openings, (3) cell walls, and (4) LCF.

Incorporating aluminum powder into geopolymer pastes produces a highly porous foam. The foam contains evenly distributed pores, as depicted in Figure 3A. Aluminum powder reacts with the strongly alkaline activator to produce hydrogen gas (H_2_) bubbles, which expand the wet paste prior to hardening. Researchers have previously studied interactions between aluminum and aqueous sodium hydroxide solutions [47]:

$$2\mathrm{Al}+{6H}_{2}O+2\mathrm{NaOH}\to {2\mathrm{NaAl}\left(\mathrm{OH}\right)}_{4}+{3H}_{2}$$

**Figure 4:**
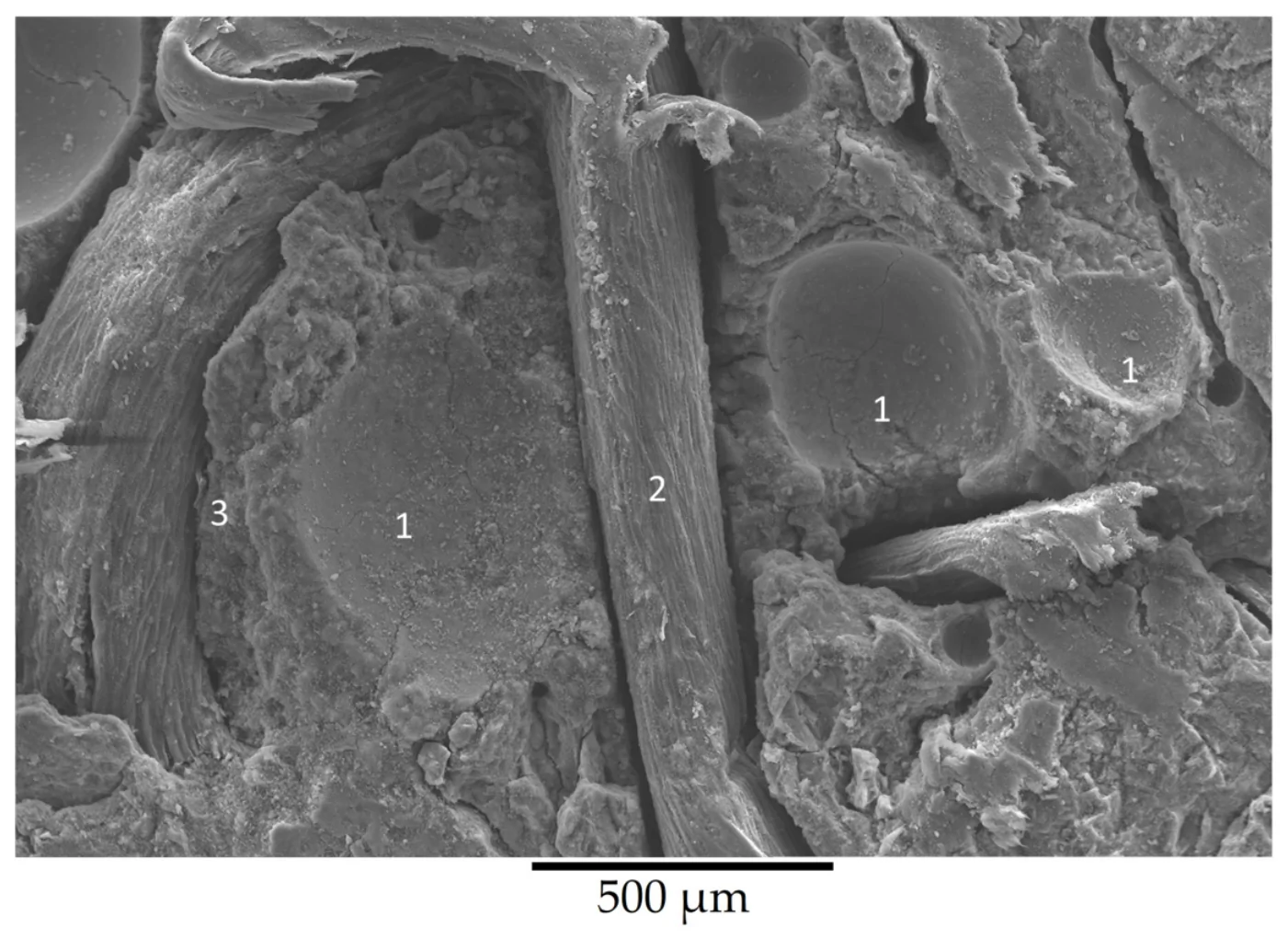
A SEM image of GLF: foam cells (1), LCF (2), and cell wall (3).

Olive oil acts as a stabilizer; when combined with aluminum powder as a foaming agent, it moderates bubble growth, refines pore-size distribution, and inhibits bubble collapse [48]. Olive oil facilitates the formation of consistent, spherical pores approximately 1 mm in diameter (Figure 3A, point 1) with well-defined cell walls (point 3). Some pores are partially open, with small openings (Figure 3, point 2). Prior studies indicate that both open and closed pores contribute to a lightweight foam with effective micropollutant absorption capabilities [38]. The incorporation of 3D LCF (Figure 3B, point 4) introduces slight modifications to the pore structure, though the overall pore size remains largely unchanged. During foaming, the cell sizes within the geopolymer composite are influenced by the interstitial spacing of the LCF mat, where thick cell walls form an interphase along the fibers, as illustrated in Figure 4. No cell wall openings were observed with the prior addition of LCF; however, microchannels may develop at the geopolymer-fiber interface.

Table 2 shows that incorporating natural fibers decreases the proportion of open cells. Water absorption, associated with the open pore system, is 270% for GF and 145% for GLF. Nonetheless, the overall bulk density after saturation remains nearly constant with the addition of LCF, recorded at 0.74 for GF and 0.76 for GLF. This density is vital to ensuring the foam remains buoyant in water for an extended period. GLF shows lower water absorption because microscopic changes in pore structure (see Figure 4, point 1) allow LCFs to interfere with pore clustering, as shown in the SEM image (Figure 4, point 2). Furthermore, the cell walls (Figure 4, point 3) are compacted at the fiber-pore interface. The SEM images confirm that the spaces between LCFs are sufficiently expansive to generate macropores.

### 3.2. Analysis of Phase Composition and Microstructure of GLF

**Figure 5:**
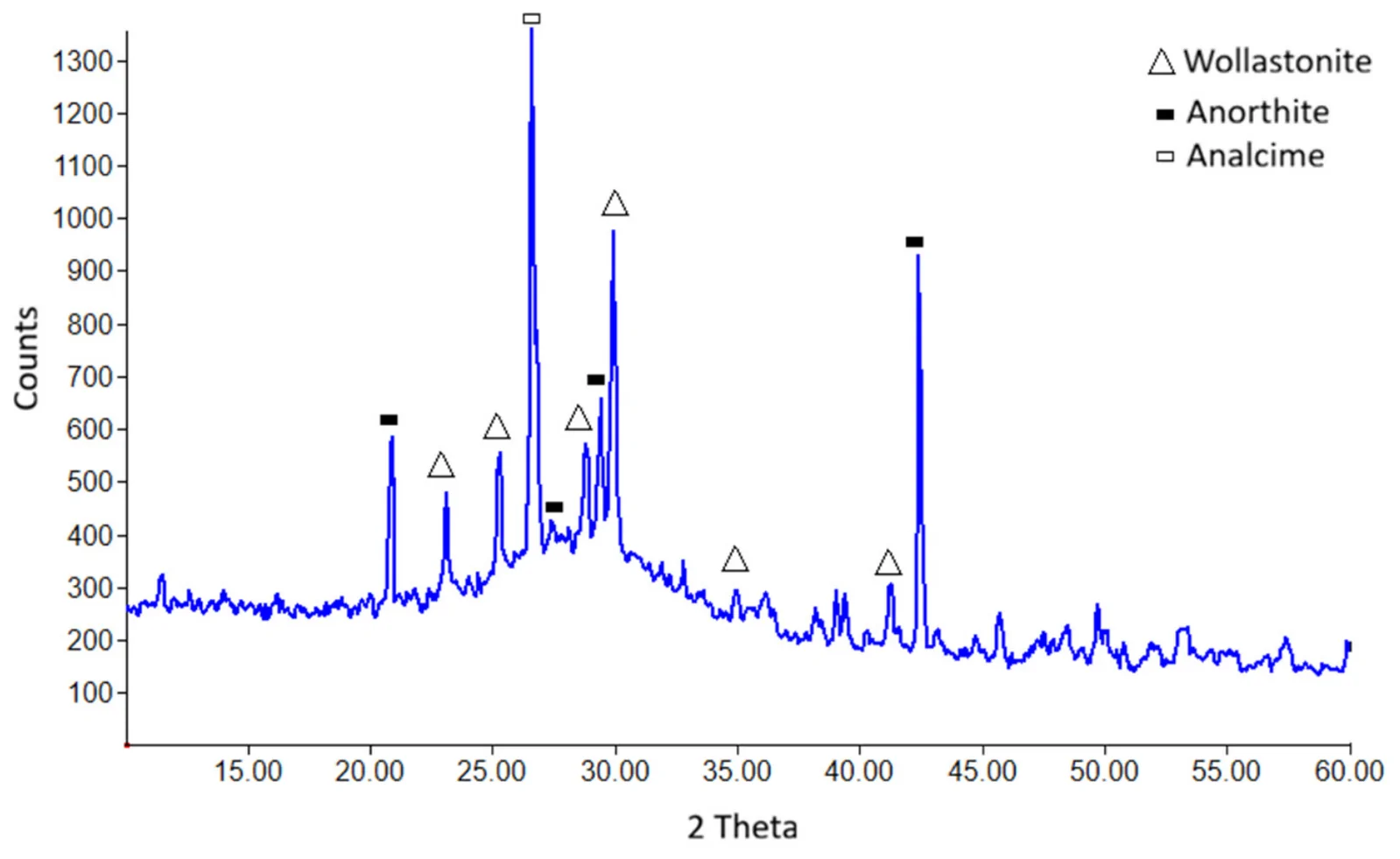
Qualitative XRD patterns for the matrix of geopolymer foam composite (GLF).

**Figure 6:**
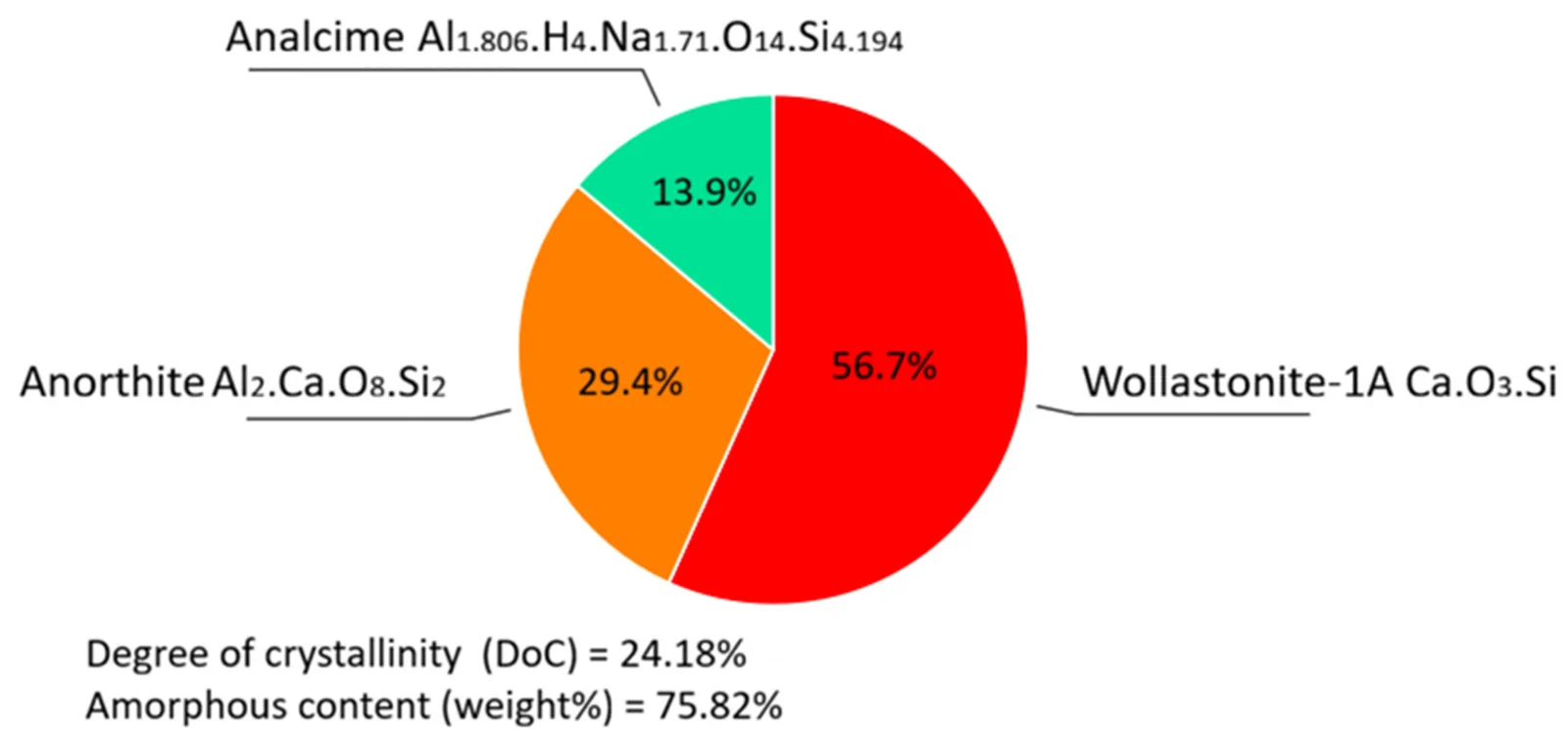
Composition of the crystalline phase of the GLF matrix.

An X-ray diffraction (XRD)analysis was performed to determine the phase composition of the GLF matrix, as shown in Figure 5. The high background signal between 20° and 40° indicated an amorphous phase. Rietveld refinement identified three crystalline phases: wollastonite, anorthite, and analcime, which accounted for 56.7%, 29.4%, and 13.9%, respectively (see Figure 6). The degree of crystallinity was 24.18%, consistent with values reported in other studies [41].

The GLF matrix shows characteristic XRD peaks of wollastonite, a precursor material. Its XRD patterns also reveal two additional crystalline phases: anorthite and analcime. Anorthite (CaAl_2_Si_2_O_8_) forms when calcium ions from wollastonite incorporate into the geopolymer matrix. As a tectosilicate mineral, it is used in water filtration [49] and improves the geopolymer’s strength. Analcime (NaAlSi_2_O_6_⋅H_2_O), a porous zeolite mineral, is used in water purification to remove heavy metals, ammonium, nitrates, and organic dyes through ion exchange and adsorption [50]. These results confirm wollastonite’s role as a reactive filler and precursor in the geopolymer matrix, alongside metakaolin.

**Figure 7:**
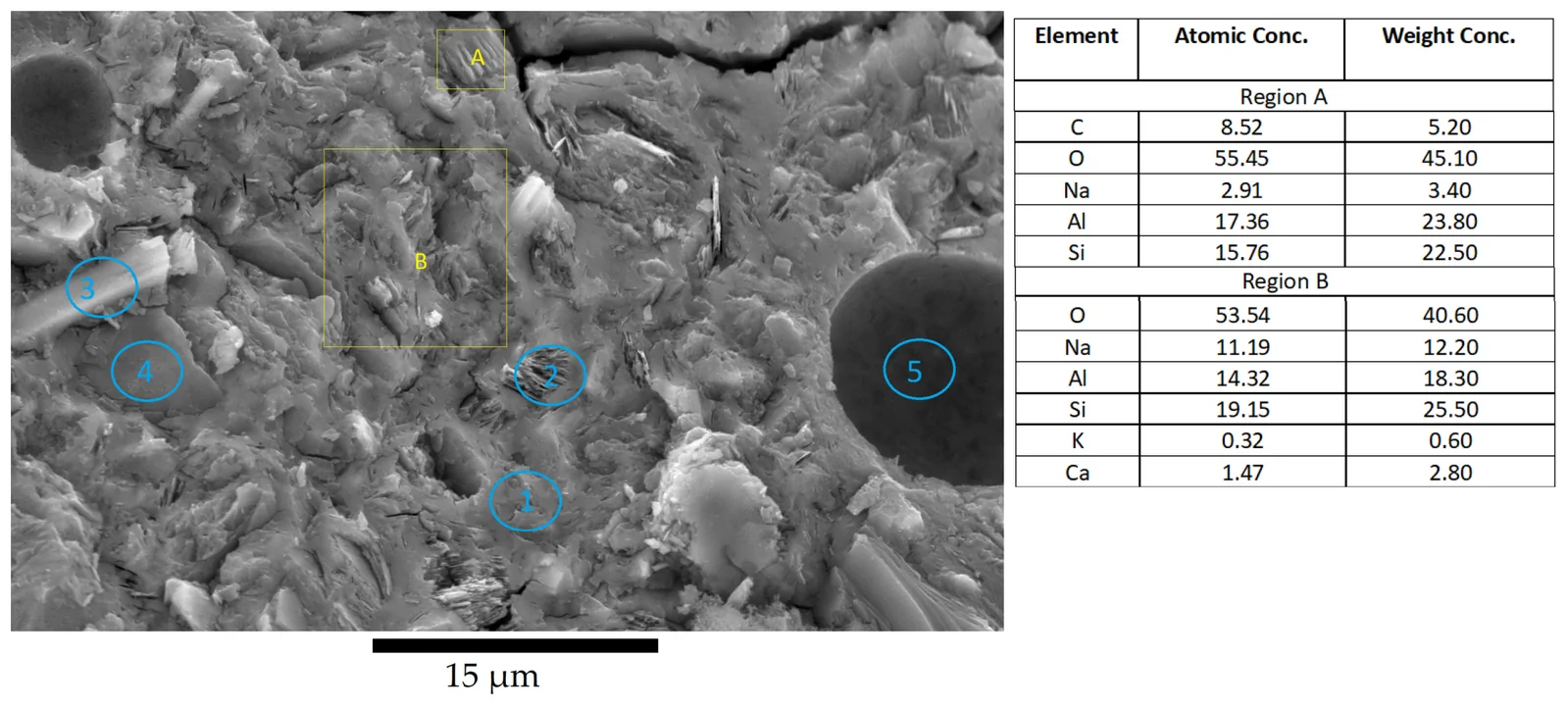
EDS-SEM image of GLF: geopolymer binder (1), metakaolin (2), wollastonite (3), silica (4), and pores (5).

Figure 7 illustrates SEM images depicting the transformation of metakaolin and wollastonite into the geopolymer matrix. In Figure 7 (point 2), residual metakaolin is observed as layered structures with gaps, indicating partial dissolution during geopolymerization. Fibrous wollastonite is visible as microfibers (Figure 7, point 3). As documented in previous studies [38], both minerals—metakaolin and wollastonite—undergo partial dissolution, forming the geopolymer binder (Figure 7, point 1). The microstructure also includes dispersed silica aggregates (Figure 7, point 4). Macropores resulting from foaming exhibit a regular spherical morphology (Figure 7, point 5).

**Figure 8:**
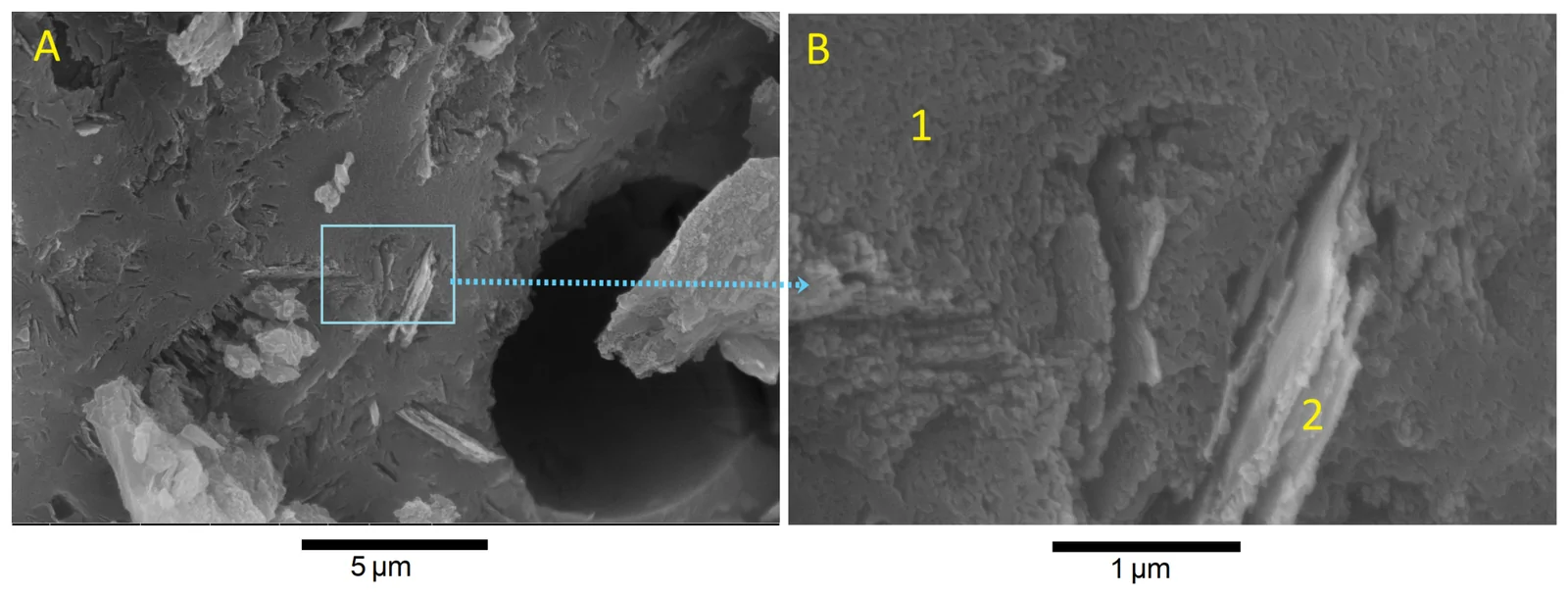
SEM images showing the mesopore network in the geopolymer foam matrix (**A**), with a higher magnification image (**B**): network of mesopores (1) and nanoparticles (2).

The geopolymer binder appears as a uniform matrix surrounding the foam’s macropores, as shown in Figure 8A. At much higher magnification in Figure 8B, a network of mesopores with approximately 50 nm pore widths is revealed (point 1). Additionally, random gaps filled with nanoparticles are visible and highlighted as point 2 in Figure 8B. This nanoscale pore network enhances water treatment capabilities due to its large surface area.

The EDS analysis of area (A) indicates that the geopolymer binder mainly comprises aluminum silicate with low sodium levels (see Figure 7). Its Si/Al molar ratio is nearly 1, similar to metakaolin. The presence of Na and C ions points to Na_2_CO_3_ formation, resulting from carbonation of residual alkaline activators. The carbon in the geopolymer foam could also originate from cellulose polymer chains (C_6_H_10_O_5_)_n_, the organic backbone of LCF. In area (B), the EDS analysis shows that the geopolymer binder forms a Na-Al-Si-Ca matrix with molar ratios of 1:1.3:1.7:0.1, aligning with previous research on Na-Al-Si binders. The calcium indicates the likely formation of a secondary Ca-Al-Si geopolymer binder.

**Figure 9:**
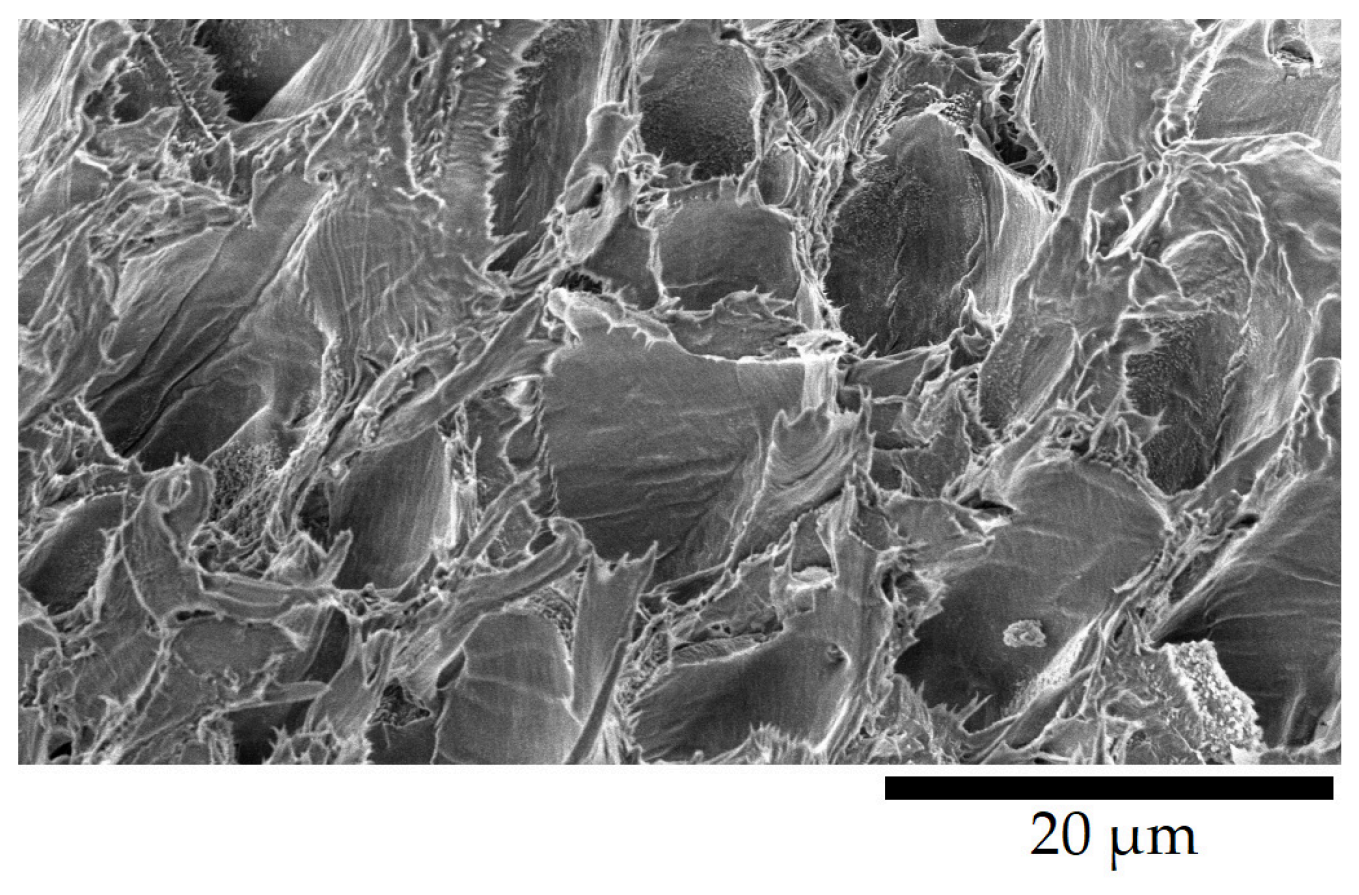
Cross-section of LCF.

In the highly alkaline setting of geopolymerization, LCF (lignin-containing lignocellulose fibrils) helps remove surface impurities, break down amorphous hemicellulose and lignin, and increase surface roughness for better bonding [51]. It also helps disassemble fiber bundles into finer microvascular fibrils (Figure 9), which significantly enhances the tensile and flexural strength of the GLF. Besides oxygen and hydrogen, lignin is about 60% carbon by weight and acts as a carbon source within the geopolymer matrix, as reported by the EDS analysis as shown in Figure 7.

### 3.3. Mechanical Characterization of the Geopolymer Foams

**Figure 10:**
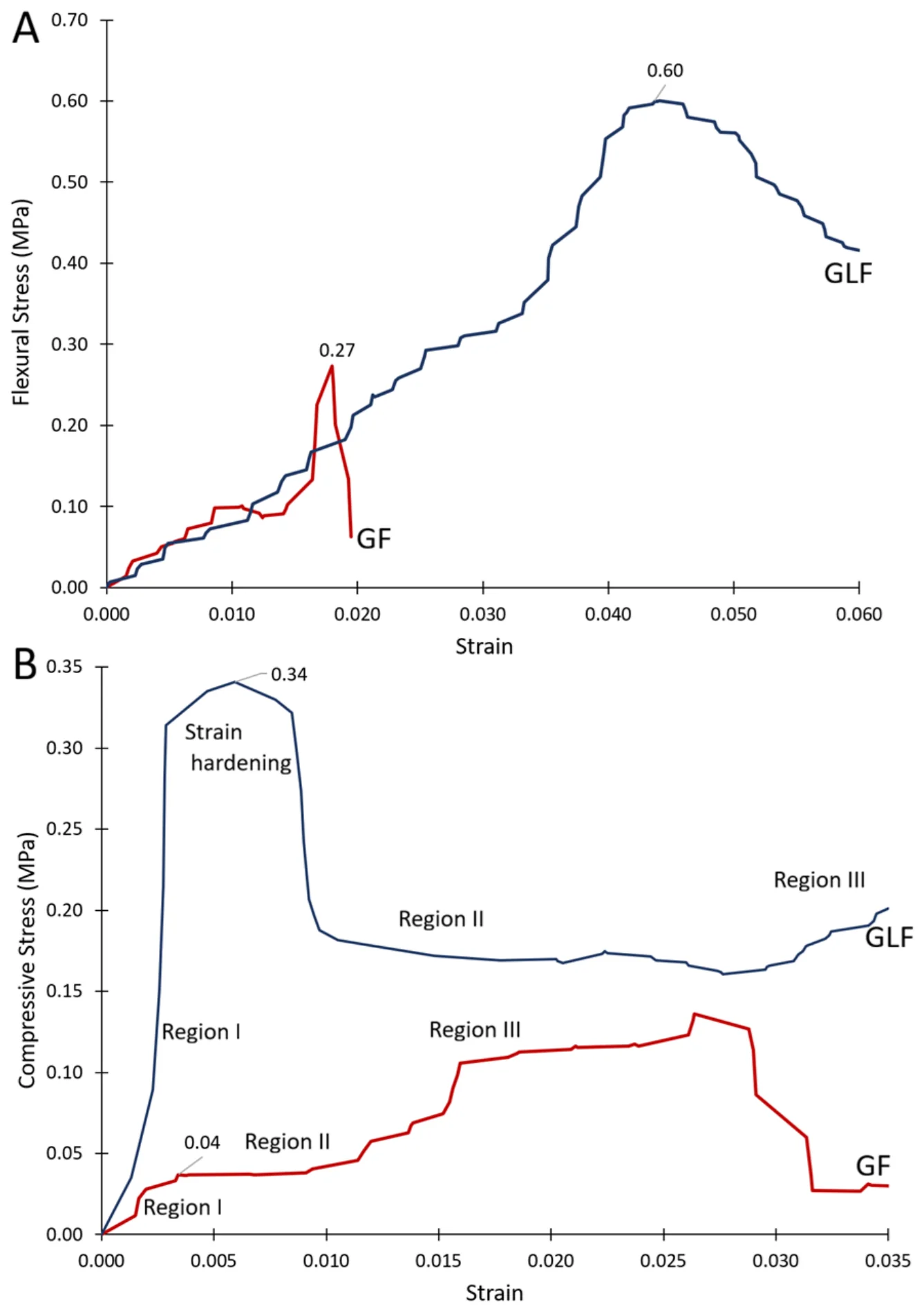
Flexural (**A**) and compressive (**B**) strengths of geopolymer foams (GF and GLF).

Figure 10A displays the measured flexural stress–strain curves for the foams. The initial slopes of GF and GLF are nearly identical, indicating similar stiffness. During flexural testing, GF failed abruptly at around 2% strain, with a low flexural strength of 0.27 MPa (Figure 10A). In contrast, the GLF generally exhibited a higher failure strain of 4.5% and a flexural strength of 0.60 MPa. Adding LCF significantly improved both stress and strain capacities, doubling the flexural strength and allowing failure at higher strain levels. This enhancement in flexural performance mainly results from embedding 3D LCF within the geopolymer foam matrix.

Figure 10B illustrates the foams’ compressive stress–strain curves. In the elastic phase, GLF exhibits a significantly higher compressive strength of 0.34 MPa than GF’s 0.04 MPa, owing to the three-dimensional LCF within the geopolymer matrix. Typical geopolymer foam compression involves three primary stages: (1) a linear elastic phase where cell walls elastically buckle; (2) a plateau representing plastic deformation and collapse at the plateau stress; and (3) densification, characterized by a sharp increase in stress as collapsed cells are compacted. The fibers primarily extend the elastic phase, as the three-dimensional LCF structure absorbs additional energy [52]. Introducing the 3D LCF results in a sharp increase in region I and strain hardening between regions I and II.

Following elastic deformation, cellular collapse initiates and progresses through the plateau region. Notably, the GLF foam’s plateau duration exceeds that of GF. As cell debris fills the remaining voids, the foam densifies, increasing its density [53]. Throughout all stages, GLF maintains the highest stress levels, indicating superior compressive strength. During testing, the stress–strain curve fluctuates as cells layer and fracture once stress surpasses the yield point of the cell walls in the geopolymer foam (Figure 10B). When cells interlock, stress rises rapidly with strain.

### 3.4. Adsorption Potential of GLF Composite for Water Treatment

#### 3.4.1. Visual Inspection of the Adsorption of MB

**Figure 11:**
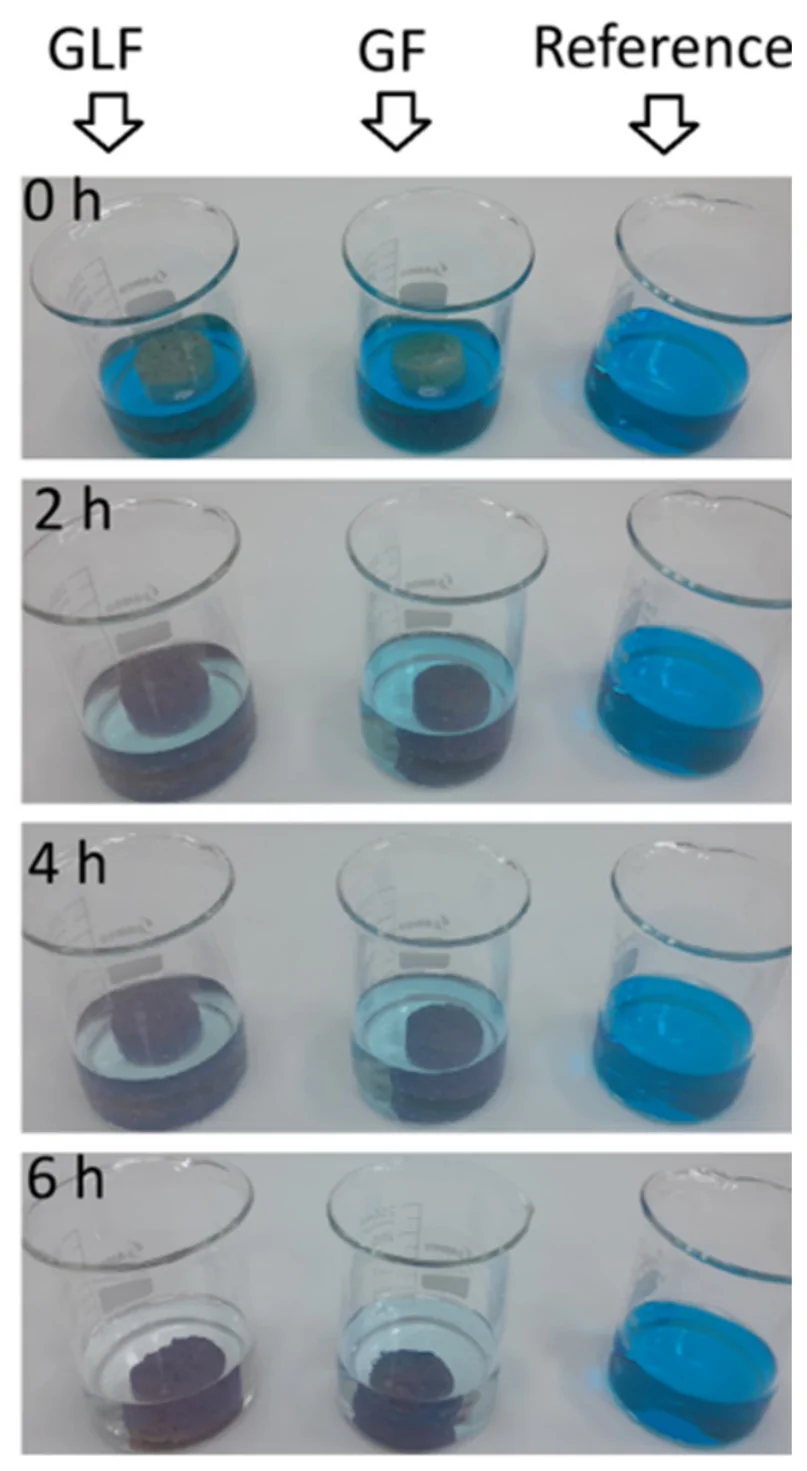
Visual evidence of MB dye adsorption on the geopolymer foams.

Figure 11 provides visual evidence of the adsorption process during the treatment of a methylene blue (MB) dye solution using geopolymer foam and a geopolymer foam composite. Specifically, the geopolymer foams were initially immersed in an MB solution. Over time, water films formed on the foams, initiating wetting. The dye concentration decreased as dye adsorbed onto the adsorbent surface. The geopolymer foam surface was negatively charged, which facilitated the attraction of positively charged dye molecules [2]. MB dye molecules saturated all available active sites on the geopolymer foams until the dye solution’s color changed from dark blue to clear. For the geopolymer foam, the white coating turned blue, confirming maximum dye adsorption. After six hours, the GLF solution became transparent, indicating complete MB removal. Although the GF solution appeared clearer over time, it remained slightly blue, suggesting that GLF’s adsorption rate surpasses that of GF. This observation implies that incorporating vascular LCF enhances MB dye adsorption (Figure 9).

GLF contains both a geopolymer matrix with a mesoporous network system and high ion exchange capacity [54], and LCF with high cellulose content. These two components play a role in water treatment. LCF, a form of vascular microfiber (Figure 9), effectively removes a variety of contaminants [55], a capacity that is enhanced when utilized with an alkaline solution during the geopolymerization process. Surface modifications of LCF in an alkaline environment during geopolymerization further enhance its pollutant-adsorption capabilities [56]. Consequently, both components of GLF contribute to water treatment, and GLF demonstrates a significantly higher capacity for water purification than GF alone, as shown in Figure 11.

#### 3.4.2. Adsorption of Metallic Ions and MB onto Geopolymer Foam Composite (GLF)

**Figure 12:**
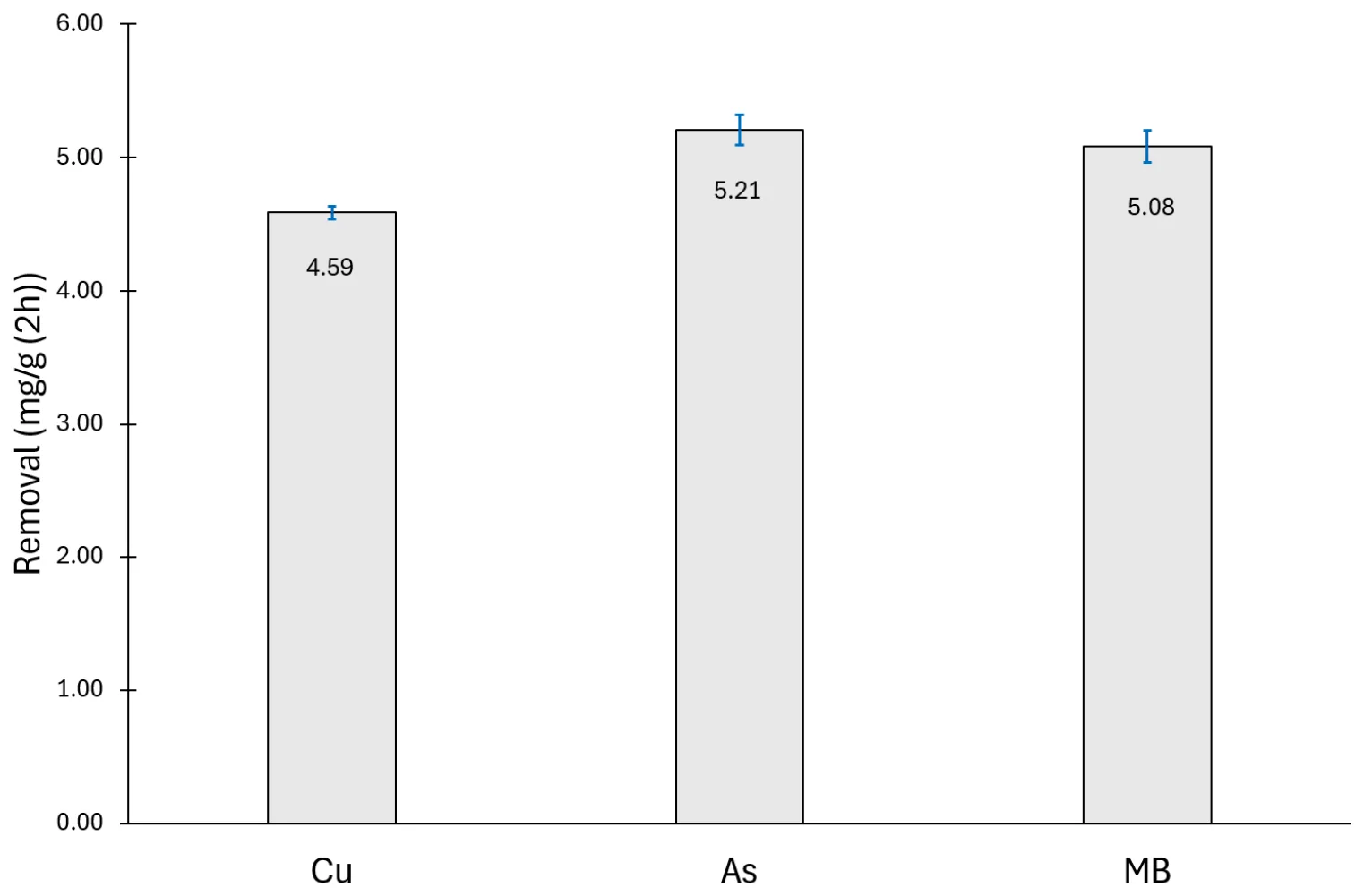
Removal of copper ions, cyanide ions, and Methylene Blue from solutions containing 75 ppm of each within 2 h for sample GLF.

The initial study examined how Cu, As, and MB ions are adsorbed onto GLF, primarily assessing GLF’s adsorption capacity without delving into the specific mechanisms, which are detailed in another study [2]. Previous research on GF also reported adsorption studies [38]. Figure 12 shows that GLF strongly adsorbed copper, arsenide ions, and MB dye. After two hours, the adsorption capacities reached 4.59 mg/g for Cu, 5.21 mg/g for As, and 5.08 mg/g for MB dye. Notably, adding LCF as a reinforcement significantly enhanced the geopolymer foam’s heavy-metal adsorption, outperforming other geopolymer foams [38] and geopolymer materials [19,20,57]. These results suggest that geopolymer foam composites effectively adsorb heavy-metal ions in acidic environments and are suitable for wastewater treatment.

**Figure 13:**
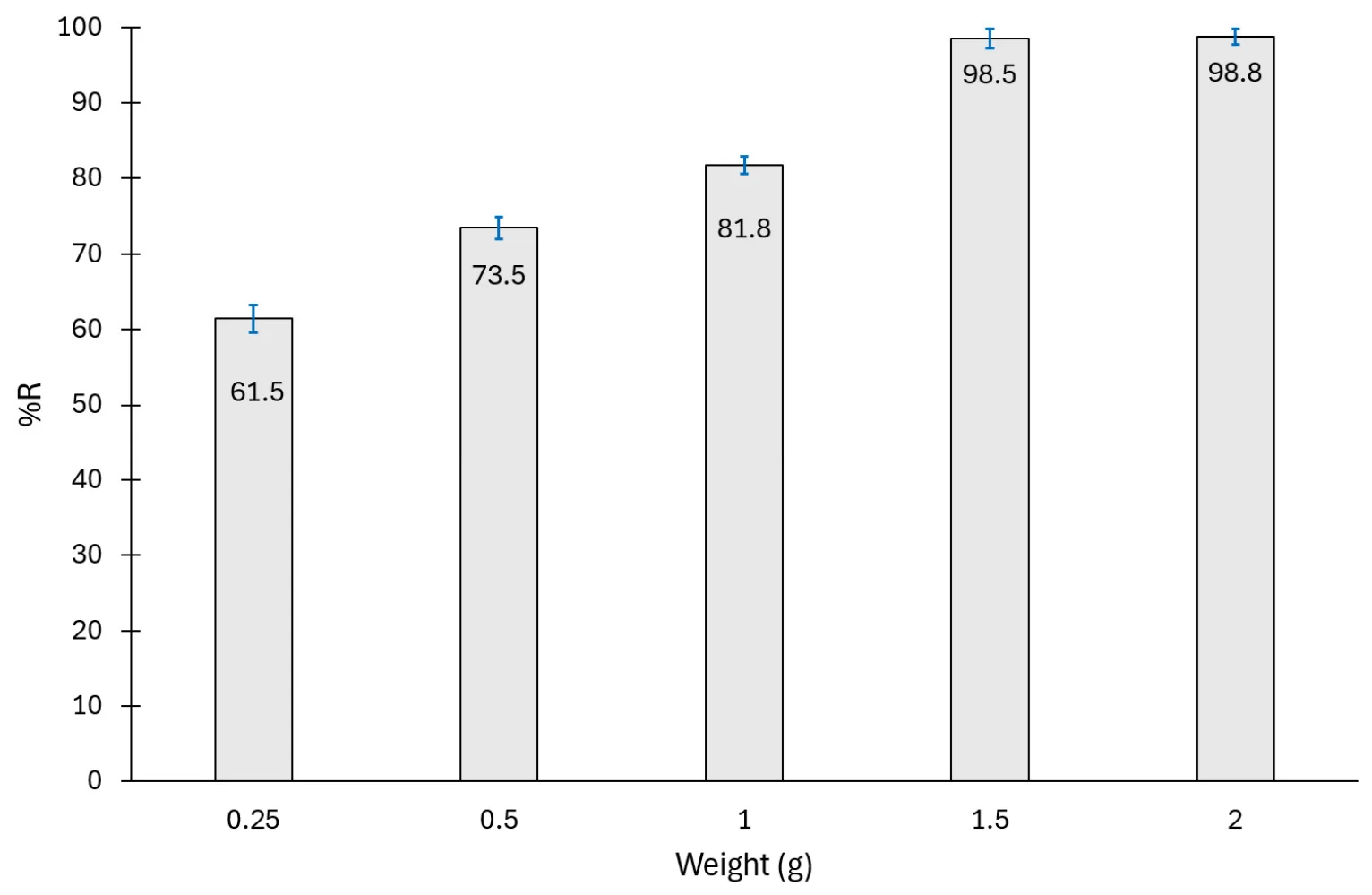
Removal of copper ions (100 ppm) from water (50 mL (solution)/g (GLF)) within 2 h.

The maximum removal efficiency by mass reached 98% (1.5 g) after two hours of contact time, as shown in Figure 13. The Cu^2+^ removal rate increased rapidly with 0.25 g of GLF, reaching 61.5%, then increased more gradually as the foam mass increased to 1.5 g. Once the GLF mass reached 1.5 g, the removal efficiency remained stable throughout the adsorption process. This pattern results from the high adsorption capacity, attributable to numerous free and active binding sites for Cu^2+^. Augmenting the GLF mass from 0.25 g to 1.5 g enhanced Cu^2+^ removal from 61% to 98.8%. At a contact duration of 120 min, the removal efficiency remained consistent even as the foam mass increased to 2 g. These results indicate that selecting the optimal foam mass can effectively remove most Cu ions from water. A comparative analysis with other materials, presented in Table 3, reveals that GLF exhibits some of the highest Cu removal efficiencies. Its performance is comparable to that of advanced materials such as Cobalt Ferrite/Activated Carbon [31] and Cellulose Nanofiber-Mesoporous Silica (CNF-MSN) Composite [33].

**Figure 14:**
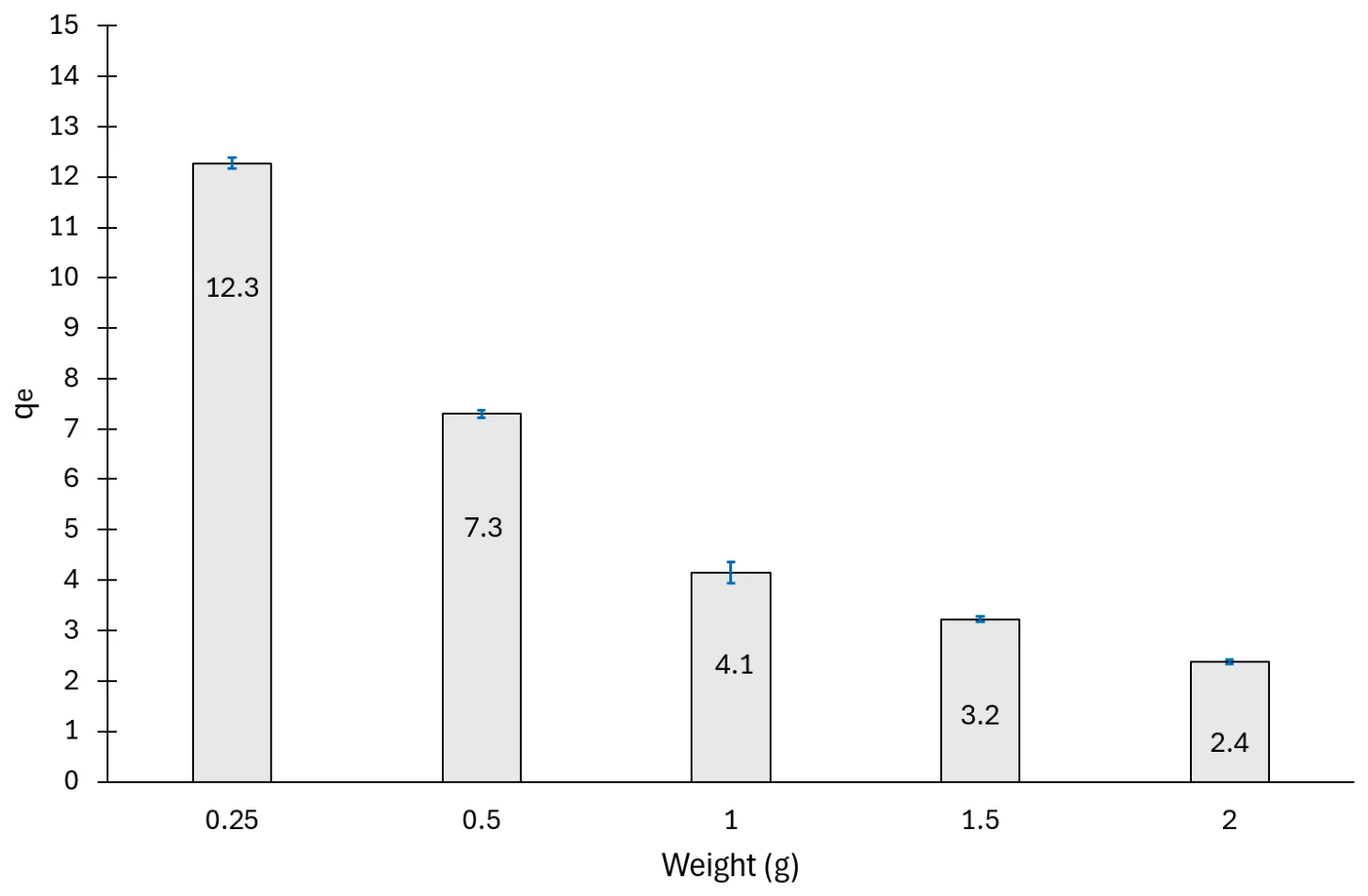
The metal uptake capacity (qe in mg/g) of GLF toward Cu ions as a function of foam mass.

The equilibrium adsorption capacity (qe) of GLF for Cu ions depends on GLF mass, as shown in Figure 14. Reducing the GLF mass increases qe. Specifically, qe rises from 2.4 mg/g to 12.3 mg/g as the GLF mass decreases from 2 g to 0.25 g. Meanwhile, the surface area increases while the volume per unit mass decreases, which may explain the increase in qe.

These findings demonstrate that GLF effectively adsorbs Cu ions and that its adsorption capacity can be precisely adjusted by varying the foam’s volume (mass). The dependence of uptake capacity on GLF volume supports technical analysis and helps select the appropriate GLF element volume for specific applications.

#### 3.4.3. Adsorption Mechanisms

Micropollutant adsorption on GLF involves multiple mechanisms. A metakaolin-based geopolymer typically has a Cation Exchange Capacity (CEC) of 1.5 to 3.0 meq/g, comparable to that of natural zeolites [65]. Each aluminum atom (AlO_4_^−^ tetrahedron) carries a negative charge that an alkali cation (Na^+^ or K^+^) must balance, though not all sites are accessible. Some alkali ions remain trapped within the dense, amorphous aluminosilicate matrix and are not exposed to the surface. Foaming the geopolymer increases its surface area, exposing more alkali ions. Positively charged heavy metal ions such as Pb, Cd, Cu, Ni, and Zn can then displace the alkali ions and bind to active surface groups, forming stable coordination bonds [66].

Wollastonite, a reactive filler within the GLF matrix (see Figure 5), partially dissolves during geopolymerization, releasing calcium ions (Ca^2+^) that neutralize excess phosphorus and increase pH. Its alkaline properties and active surface sites also aid in adsorbing heavy metals [67]. Following the release of calcium ions, wollastonite converts into a calcium-sodium silicate hydrate gel (see Figure 7). The resulting amorphous silicate formed during the dissolution of both wollastonite and metakaolin shows high removal efficiency, removing up to 98% of toxic divalent and trivalent metal ions, including Cd^2+^, Zn^2+^, Cu^2+^, Ni^2+^, Pb^2+^, and Cr^3+^ [68]. The GLF matrix (Figure 8) has pores about 50 nm in size and physically traps contaminants by encasing micropollutants within the fixed-size pores and the material’s amorphous matrix.

LCF Cellulose is completely biodegradable, renewable, and non-toxic. Its dense microfibrous networks capture sediments, organic matter, and microorganisms as water passes through its porous structure [69]. Reactive groups with high surface area, such as hydroxyl and carboxyl groups, attach to and remove toxic chemicals, dyes, and pharmaceutical waste.

In summary, the GLF composite effectively removes pollutants due to active contaminant extraction by both the matrix and fibers. Adding wollastonite as a precursor or filler improves micropollutant removal, while employing vascular LCF as continuous microtube fibers increases the composite’s ability to adsorb heavy metals and organic pollutants.

## 4. Conclusions

This study aimed to synthesize and analyze a geopolymer foam composite reinforced with 3D LCF. The gaps between the LCF, as a cellular natural fiber, facilitate foaming and ensure strong adhesion with the foam. Consequently, the composite’s flexural strength doubled, and its compressive strength grew eightfold. Unlike non-reinforced foam, this new composite demonstrates high mechanical performance suitable for water treatment and various engineering uses. Microscopic features showed minimal changes, with a reduction in macropore size due to pressure from the LCF cells. Key microstructural phases include residual precursors (metakaolin, wollastonite) and geopolymer binder (amorphous phase, anorthite, and analcime). Anorthite formation confirms the incorporation of wollastonite as a precursor/reactive filler and a source of Ca ions during the geopolymerization. The geopolymer binder exhibits a mesopore network which assists in water treatment. The foam contains both closed and open pores; closed pores help maintain bulk density and buoyancy during saturation, while open pores enhance water absorption for purification. A comparison with non-reinforced geopolymer foam highlights these properties. The study indicates LCF significantly boosts the foam’s adsorption capacity. LCF-modified foam shows a 98% adsorption rate for Cu ions and a significant increase in MB dye adsorption. GLF retains a low bulk density even when saturated, demonstrating its suitability for manufacturing floating, permeable bulk materials. Overall, this research opens exciting possibilities for developing high-performance geopolymer foams with enhanced mechanical and adsorption features.

## Figures and Tables

**Table 1:** Mixes and compositions of the prepared geopolymer foams.

	Metakaolin (g)	Wollastonite (g)	Na_2_SiO_3_ (g)	NaOH (g)	LCF (g)	Olive Oil (mL)	Aluminum Powder (g)
GF	100	25	100	25	0	1	0.4
GLF	100	25	100	25	8	1	0.4

**Table 2:** Physical properties of the geopolymer foams.

	Bulk Density (Dry) ρ g/cm^3^	Bulk Density (Saturation) ρ_sat_ g/cm^3^	Water Absorption Ww wt.%
GF	0.20	0.74	270
GLF	0.31	0.76	145

ρ: bulk density (mass per bulk volume); ρ_sat_: saturated density (mass after water absorption divided by bulk volume); and Ww: water absorption (the weight of water absorbed by the material in the saturated state divided by the dry weight).

**Table 3:** Maximum removal percentage (R%) of copper (Cu) ions for different materials, based on previous studies, although testing conditions may vary.

Material	R%	pH	References
Geopolymer foam composite (GFL)	98	5	Current study
Cobalt ferrite/activated carbon (COF/AC) composite	97.45	5	[58]
Polyester-Kaolin Composite	45.7	7	[59]
Cellulose Nanofiber-Mesoporous Silica (CNF-MSN) Composite	97.85	4–6	[60]
Limestone	95	8.5	[61]
Modified Biochars	93–98.3	5.5–6.0	[62]
Raw Banana Leaves (BL) Adsorbent	60–90	4–6	[63]
Nano Sawdust	93	6.5–7	[64]
Magnetite Nano-Adsorbent	62.61	5–6	[45]

## Data Availability

The original contributions presented in this study are included in the article. Further inquiries can be directed to the corresponding author.
